# Evaluation of Immunohistochemical Markers, CK17 and SOX2, as Adjuncts to p53 for the Diagnosis of Differentiated Vulvar Intraepithelial Neoplasia (dVIN)

**DOI:** 10.3390/ph14040324

**Published:** 2021-04-02

**Authors:** Shatavisha Dasgupta, Senada Koljenović, Thierry P. P. van den Bosch, Sigrid M. A. Swagemakers, Nick M. A. van der Hoeven, Ronald van Marion, Peter J. van der Spek, Helena C. van Doorn, Folkert J. van Kemenade, Patricia C. Ewing-Graham

**Affiliations:** 1 Department of Pathology, Erasmus MC, University Medical Centre Rotterdam, 3015 GD Rotterdam, The Netherlands; s.koljenovic@erasmusmc.nl (S.K.); t.vandenbosch@erasmusmc.nl (T.P.P.v.d.B.); s.swagemakers@erasmusmc.nl (S.M.A.S.); r.vanmarion@erasmusmc.nl (R.v.M.); p.vanderspek@erasmusmc.nl (P.J.v.d.S.); f.vankemenade@erasmusmc.nl (F.J.v.K.); p.ewing@erasmusmc.nl (P.C.E.-G.); 2 Department of Clinical Bioinformatics, Erasmus MC, University Medical Centre Rotterdam, 3015 GD Rotterdam, The Netherlands; 3 Department of Gynecology and Obstetrics, Erasmus MC, University Medical Centre Rotterdam, 3015 GD Rotterdam, The Netherlands; n.vanderhoeven@erasmusmc.nl; 4 Department of Gynecologic Oncology, Erasmus MC Cancer Institute, University Medical Centre Rotterdam, 3015 GD Rotterdam, The Netherlands; h.vandoorn@erasmusmc.nl

**Keywords:** vulva, keratin-17, SOX2 protein, human, carcinoma-in-situ, neoplasms, squamous cell neoplasm

## Abstract

Histological diagnosis of differentiated vulvar intraepithelial neoplasia (dVIN), the precursor of human papillomavirus (HPV)-independent vulvar squamous cell carcinoma (VSCC), can be challenging, as features of dVIN may mimic those of non-dysplastic dermatoses. To aid the diagnosis, p53-immunohistochemistry (IHC) is commonly used, and mutant expression patterns are used to support a histological diagnosis of dVIN. However, a proportion of dVIN can show wild-type p53-expression, which is characteristic of non-dysplastic dermatoses. Furthermore, recent research has identified a novel precursor of HPV-independent VSCC—the p53-wild-type differentiated exophytic vulvar intraepithelial lesion (de-VIL). Currently, there are no established diagnostic IHC-markers for p53-wild-type dVIN or de-VIL. We evaluated IHC-markers, cytokeratin 17 (CK17), and SRY-box 2 (SOX2), as diagnostic adjuncts for dVIN. For this, IHC-expression of CK17, SOX2, and p53 was studied in dVIN (*n* = 56), de-VIL (*n* = 8), and non-dysplastic vulvar tissues (*n* = 46). For CK17 and SOX2, the percentage of cells showing expression, and the intensity and distribution of expression were recorded. We also performed next generation targeted sequencing (NGTS) on a subset of dVIN (*n* = 8) and de-VIL (*n* = 8). With p53-IHC, 74% of dVIN showed mutant patterns and 26% showed wild-type expression. Median percentage of cells expressing CK17 or SOX2 was significantly higher in dVIN (p53-mutant or p53-wild-type) and de-VIL than in non-dysplastic tissues (*p* < 0.01). Diffuse, moderate-to-strong, full epithelial expression of CK17 or SOX2 was highly specific for dVIN and de-VIL. With NGTS, *TP53* mutations were detected in both dVIN and de-VIL. We infer that immunohistochemical markers CK17 and SOX2, when used along with p53, may help support the histological diagnosis of dVIN.

## 1. Introduction

Vulvar squamous cell carcinoma (VSCC) is classified etiologically into human papilloma virus (HPV)-related, and HPV-independent subtypes [1,2,3,4]. HPV-independent VSCC is the more prevalent subtype [3] that typically arises in the setting of chronic dermatoses. Differentiated vulvar intraepithelial neoplasia (dVIN) is the most well-characterized precursor lesion of HPV-independent VSCC [1,2,3,4]. Studies report that dVIN can progress rapidly to VSCC [5,6]; therefore, lesions diagnosed on histology as dVIN are surgically excised [7,8]. However, histological diagnosis of dVIN can be difficult even for experienced pathologists [2], and may suffer from suboptimal reproducibility [9,10].

The difficulty in diagnosing dVIN stems largely from its subtle histological appearance, which may mimic that of reactive/non-dysplastic dermatoses [11,12,13,14]. Therefore, to accurately discriminate dVIN from non-dysplastic dermatoses, p53-immunohistochemistry (IHC) is commonly used as an ancillary tool [12,13,15]. Mutant patterns of p53-expression are used to support a histological diagnosis of dVIN, as these have been reported to reflect *TP53* mutations that characterize dVIN [16,17,18]. However, 17–42% of dVIN can show wild-type p53-expression, which is usually observed in non-dysplastic lesions [19,20,21,22]. Furthermore, recent research has identified a novel putative precursor of HPV-independent VSCC, named differentiated exophytic vulvar intraepithelial lesion (de-VIL) [23]. de-VILs are acanthotic or verruciform lesions lacking sufficient histological atypia for the diagnosis of dVIN, and on IHC, show wild-type p53-expression [23,24,25]. There is a need for IHC markers that may facilitate the diagnosis of precursors of HPV-independent VSCC, in particular, the p53-wild-type lesions [17,26].

In recent years, multiple studies have investigated immunohistochemical expression of cytokeratin 17 (CK17) and SRY-box transcription factor 2 (SOX2), in precursors of genital and non-genital SCCs [20,27,28,29,30,31,32,33,34,35]. CK17 is a differentiation marker that maintains cellular organization [36], and SOX2 is a stemness regulator that maintains self-renewal properties of normal and malignant cells [36]. CK17 and SOX2 have been reported to show increased expression in squamous cell carcinoma (SCC) precursors compared to normal tissues, and both have been deemed as promising diagnostic markers [20,27,33,34,35].

In this study, we evaluated immunohistochemcial markers CK17 and SOX2 as adjuncts to p53 for the diagnosis of dVIN. For this, we examined the expression of these markers in dVIN, de-VIL, high-grade squamous intraepithelial lesion (HSIL; precursor of HPV-related VSCC), and non-dysplastic vulvar tissue. In addition, to facilitate the identification of other potential diagnostic markers, we performed next generation targeted sequencing (NGTS) on a subset of dVIN and de-VIL.

## 2. Results

### 2.1. Histology Review

A total of 137 lesions were included after histology review, comprising dVIN (*n* = 56), de-VIL (*n* = 8), HSIL (*n* = 27), and non-dysplastic vulvar tissue (*n* = 46). Non-dysplastic tissues comprised lichen sclerosus (*n* = 15), non-specific reactive changes/inflammation (*n* = 18), and histologically normal vulvar tissue (*n* = 13). Six of the histologically normal vulvar tissues were identified from resection specimens of VSCC, and 7 were residual tissues from vulvar surgeries conducted for benign pathologies in women with no history of VIN or VSCC. Clinico-pathological characteristics are presented in Table 1.

### 2.2. Immunohistochemistry (IHC)

**Figure 1:**
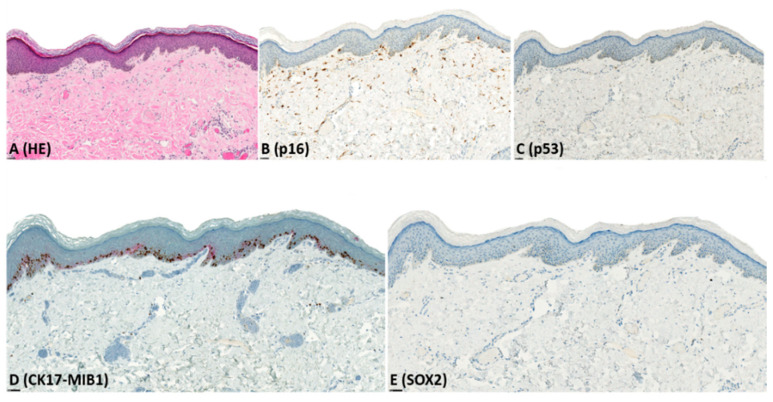
Example of immunohistochemical expression of p16, p53, CK17, MIB1, and SOX2 in li-chen sclerosus (LS); (**A**) Histological appearance of LS (hematoxylin-eosin (HE) stain); (**B**) complete lack of p16-expression; (**C**) wild-type (scattered) p53-expression; (**D**) focal, weak, CK17-expression and increased MIB1 expression; (**E**) complete lack of SOX2-expression.

**Figure 2:**
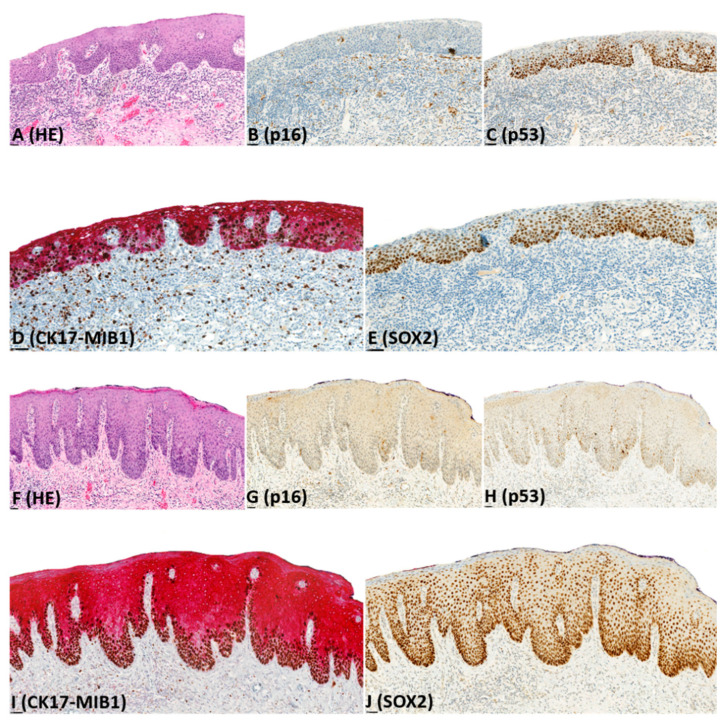
Example of immunohistochemical expression of p16, p53, CK17, MIB1, and SOX2 in differentiated vulvar intraepithelial neoplasia (dVIN); (**A**) Histological appearance of dVIN (HE-stain); (**B**) complete lack of p16-expression; (**C**) mutant pattern (basal and parabasal overexpression) of p53-expression; (**D**) diffuse, strong CK17-expression across full epithelial thickness and increased MIB1-expression; (**E**) diffuse, strong SOX2-expression across full epithelial thickness; (**F**) Histological appearance of dVIN (HE-stain); (**G**) complete lack of p16-expression; (**H**) wild-type (scattered) p53-expression; (**I**) diffuse, strong CK17-expression across full epithelial thickness and increased MIB1-expression; (**J**) diffuse, strong SOX2-expression across full epithelial thickness.

**Figure 3:**
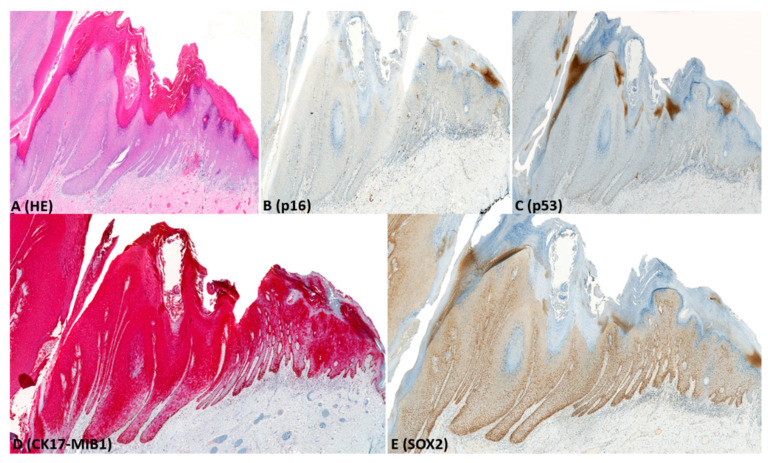
Example of immunohistochemical expression of p16, p53, CK17-MIB1, and SOX2 in differentiated exophytic vulvar intra-epithelial lesion (de-VIL); (**A**) Histological appearance of de-VIL (HE-stain); (**B**) complete lack of p16-expression; (**C**) wild-type (scattered) p53-expression; (**D**) diffuse, strong CK17-expression across full epithelial thickness and increased MIB1-expression; (**E**) diffuse, strong SOX2-expression across full epithelial thickness.

**Figure 4:**
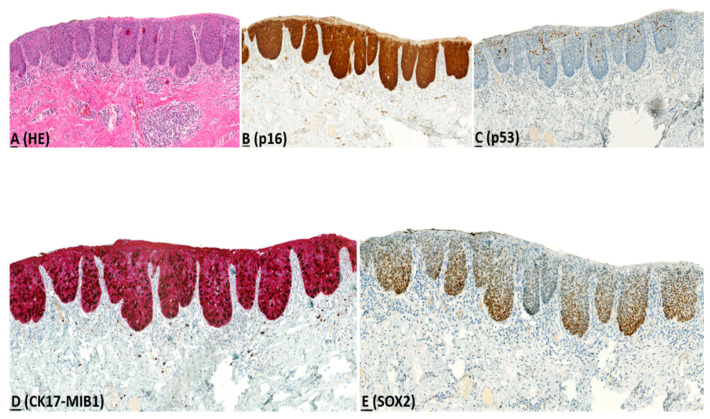
Example of immunohistochemical expression of p16, p53, CK17-MIB1, and SOX2 in high grade squamous intraepithelial lesion (HSIL); (**A**) Histological appearance of HSIL (HE-stain); (**B**) block-type p16-expression; (**C**) wild-type (mid-epithelial) p53-expression; (**D**) diffuse, strong CK17-expression across full epithelial thickness and increased MIB1-expression; (**E**) diffuse, strong SOX2-expression across full epithelial thickness.

Immunohistochemistry results are presented in Table 2, illustrated in Figure 1, Figure 2, Figure 3 and Figure 4, and summarized below.

p16: Of HSIL, 96% showed block-type, and 4% showed non-block-type expression. Block-type expression is considered to be a reliable surrogate marker of high-risk HPV-infection [37], and this was not seen in any dVIN, de-VIL, or non-dysplastic vulvar tissues.

p53: Of dVIN, 74% showed mutant patterns, and 26% showed wild-type (scattered) expression. All de-VIL showed wild-type (scattered) expression. Of HSIL, 96% showed wild-type (mid-epithelial) expression; this included the lesion that showed non-block-type p16-expression. Of non-dysplastic vulvar tissue, 83% showed wild-type (scattered) and 17% showed mutant patterns of expression.

MIB1: Increased MIB1 expression was noted in 52% of dVIN, 88% of de-VIL, all HSILs, and 39% of non-dysplastic vulvar tissues. For dVIN, de-VIL, and non-dysplastic lesions, increased MIB1 expression was most frequently observed in the basal and/or parabasal layers, whereas, for HSIL, this was across the full epithelial thickness. Difference in MIB1 expression in dVIN and non-dysplastic vulvar was not significant (*p* = 0.08).

CK17: For dVIN and de-VIL, the median percentage of cells expressing CK17 was significantly higher (*p* < 0.01) compared to non-dysplastic vulvar tissues (Figure 1, Figure 2, Figure 3 and Figure 4). Increased CK17-expression was recorded for dVIN that showed mutant pattern or wild-type p53-expression, with no significant difference among these sub-groups, in comparison with non-dysplastic vulvar tissue.

Of dVIN, 80% showed diffuse, moderate-to-strong expression, either across full epithelial thickness or in the suprabasal layers. This pattern of expression was also noted in 88% of de-VIL and 63% of HSIL. In dVIN, de-VIL, and HSIL, expression of CK17 followed the distribution of dysplastic cells. Of non-dysplastic vulvar tissue, 80% showed either no expression, or patchy, weak, expression in the suprabasal layers.

SOX2: For dVIN and de-VIL, the median percentage of cells expressing SOX2 was significantly higher (*p* < 0.01) compared to non-dysplastic vulvar tissue (Figure 1, Figure 2, Figure 3 and Figure 4). Increased SOX2-expression was recorded for dVIN that showed mutant pattern or wild-type p53-expression, with no significant difference among these sub-groups, in comparison with non-dysplastic vulvar tissue.

Of dVIN, 86% showed diffuse, moderate-to-strong expression, either across full epithelial thickness or in the basal and suprabasal layers. This pattern of expression was also noted in 88% of de-VIL and 88% of HSIL. In dVIN, de-VIL, and HSIL, the expression of SOX2 followed the distribution of dysplastic cells. Of non-dysplastic vulvar tissue, 81% showed either no expression, or scattered, weak, expression in the basal (predominantly) and suprabasal layers.

**Figure 5:**
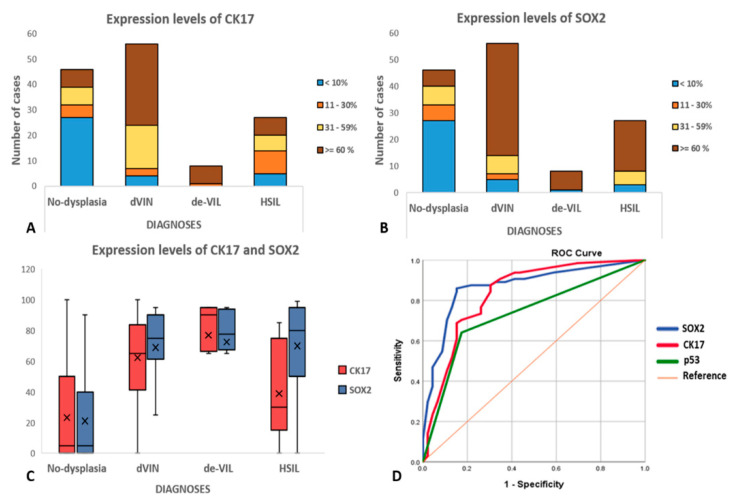
(**A**) Bar-charts depicting the levels of CK17-expression and (**B**) SOX2-expression. For each diagnostic category, color-coding represents the percentage of cells showing cytoplasmic CK17-expression, or nuclear SOX2-expression stratified as <10%, 11–30%, 31–59%, and ≥60%; (**C**) Boxplots depicting the distribution of CK17 and SOX2 expression for each diagnostic category; horizontal lines in the boxes represent the medians, the cross-marks represent the mean, and the whiskers represent the 5th and 95th percentiles; (**D**) Receiver operating characteristics (ROC) curves for SOX2 (blue line), CK17 (red line), and p53 (green line) immunohistochemistry for the diagnosis of dVIN. Area under the curve (AUC) for SOX2 = 0.87, CK17 = 0.82, and p53 = 0.78.

Receiver operating characteristic curve (ROC), sensitivity, specificity, positive predictive value (PPV), and negative predictive value (NPV): For the diagnosis of dVIN, area under the curve (AUC) for p53 was 0.78 (95% CI: 0.69–0.87); for CK17, this was 0.82 (95% CI: 0.74–0.91), and for SOX2, this was 0.87 (95% CI: 0.79–0.94) (Figure 5). SOX2 showed the highest sensitivity (86%; 95% CI: 77.6–92.1%) for the diagnosis of dVIN, whereas p53 showed the highest specificity (83%; 95% CI: 73.4–89.5%). SOX2 also showed the highest PPV (82%; 95%CI: 74.9–87.2%) and NPV (85%; 95% CI: 77.9–90.5%) for the diagnosis of dVIN (detailed in Table S1).

### 2.3. Next Generation Targeted Sequencing

**Figure 6:**
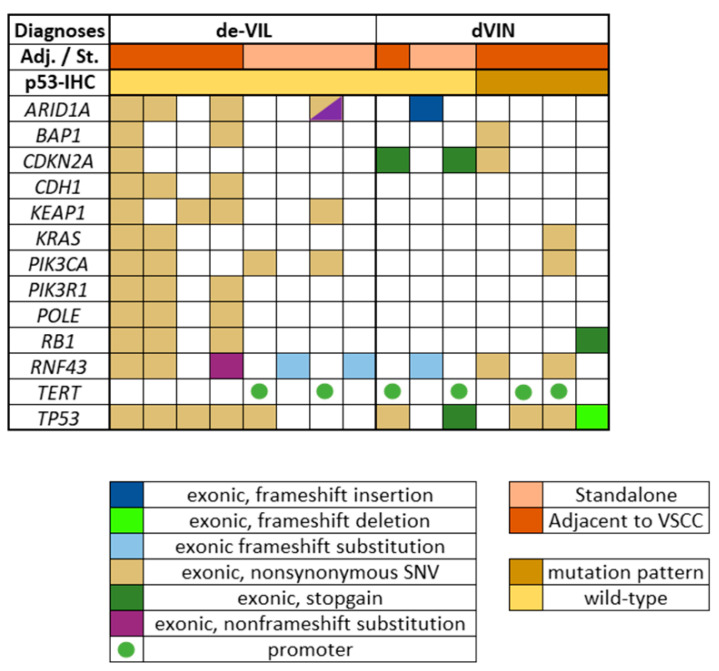
Pathogenic somatic mutations detected in at least 3 samples of de-VIL and dVIN; color-coding represents the types of mutations, p53-immunohistochemistry (IHC) results, and whether the lesion was standalone or present in tissue adjacent to vulvar squamous cell carcinoma (VSCC); Adj.: adjacent; St.: standalone; SNV: single nucleotide variation.

In the subset of dVIN (*n* = 8) and de-VIL (*n* = 8) that was studied, a total of 106 pathogenic mutations in 44 genes were identified (Table S2). The median number of mutations detected in dVIN was 3 (range: 1–6), and in de-VIL was 5 (range: 1–31). Pathogenic mutations detected in at least 3 samples of dVIN or de-VIL are depicted in Figure 6. The most commonly detected mutations for both diagnoses are described below.

dVIN: For all dVIN, mutations in *TP53* (63%), *TERT*-promoter (50%), *CDKN2A* (38%), and *RNF43* (38%) were most frequently detected. For p53-wild-type dVIN (*n* = 4), these were *TP53* (50%) and *CDKN2A* (50%), while for p53-mutant dVIN (*n* = 4), these were *TP53* (75%) and *RNF43* (50%).

de-VIL: For de-VIL, *RNF43* (63%), *TP53* (63%), *PIK3CA* (50%), *ARID1A* (50%), and *KEAP1* (50%) were most frequently detected. Mutations in *KEAP1* (50%), *CDH1* (38%)*, PIK3R1* (38%), and *POLE* (38%) were exclusively detected in de-VIL, i.e., not detected in dVIN.

## 3. Discussion

Since pathological diagnoses inform treatment decisions, accurate distinction of dVIN from reactive/non-dysplastic lesions is crucial. We observed that the majority of dVIN show diffuse, moderate-to-strong expression of CK17 and SOX2, across the full epithelial thickness or in the suprabasal layers. In contrast, the majority of non-dysplastic lesions show either no expression, or patchy/scattered weak expression of CK17 and SOX2. We therefore infer that IHC with CK17 and SOX2 can help distinguish dVIN from non-dysplastic lesions.

Recent studies have increasingly recognized that accurate diagnosis of dVIN may not be achieved on the basis of histological assessment alone [38,39,40,41]. The spectrum of histological features of dVIN can overlap with that of non-dysplastic lesions, and occasionally, with that of HPV-related HSILs as well [40,41]. Therefore, to improve diagnostic accuracy, an IHC panel comprising p16 and p53 is being recommended for lesions with a histological suspicion of dVIN [2,4,17,39]. Block-type p16-expression is a reliable surrogate of high-risk HPV-infection, and helps discriminate HSIL from dVIN [40,41]. In addition, mutant patterns of p53-expression, i.e., basal and/or parabasal over-expression, or complete absence of expression, can help distinguish dVIN from non-dysplastic lesions [42,43]. Furthermore, the mid-epithelial wild-type p53-expression, which is considered characteristic of HSIL (seen in 96% of HSIL in the current study), can also help discriminate HSIL from dVIN [44].

However, similarly to most IHC-markers, p53 is not without limitations. Increased p53-expression may not only result from the mutated protein, but also from the accumulation of the wild-type protein in response to DNA-damage in inflammatory conditions [42,45]. Therefore, increased p53-expression may be observed in non-dysplastic lesions as well [45]. Moreover, a proportion of dVIN (26% in our series), and the recently recognized VSCC precursor, de-VIL, show wild-type p53-expression [2,10,23,24]. In addition to p53 and p16, the proliferation marker MIB1 is commonly used to aid the diagnosis of dVIN. However, increased MIB1 expression can be seen both in dVIN and in reactive, non-dysplastic lesions [46,47].

Although over the past years, a number of novel diagnostic IHC-markers have been studied for dVIN, none have so far been translated to the clinics [15]. This is largely because most of these markers were evaluated in small cohorts and/or in single studies [15].

For dVIN, CK17 is the only IHC-marker that has been evaluated in more than one study, which includes one of our previous studies [20,27]. CK17 is a basal/myo-epithelial cell-associated protein [48]. In normal skin (genital/non-genital), CK17 is expressed only in the appendages, and not in the squamous epithelial lining [33,49]. Increased CK17-expression across full-epithelial thickness or in the suprabasal layers has been observed in SCC, and precursors of SCC, of the cervix [49], vulva [20,27], anus [33], larynx [34], and oral cavity [30].

SOX2 is another IHC-marker that has been widely studied in SCC of both genital and non-genital locations [28,29,32]. A recent meta-analysis identified SOX2 as a promising biomarker for tongue SCC, which is also an HPV-independent SCC [35]. SOX2 is a transcription factor postulated to promote cancer development and progression, and increased SOX2-expression has been observed in SCC, and precursors of SCC, of the cervix [50], vulva [28,29], esophagus [51], and oral cavity [52].

To evaluate CK17 and SOX2 as diagnostic adjuncts for dVIN, we systematically examined their expression in a carefully selected set of dVIN, de-VIL, HSIL, and non-dysplastic vulvar tissues. Increased expression of CK17 was observed in dVIN showing mutant patterns or wild-type p53-expression, compared to non-dysplastic vulvar tissue, in line with previous reports [20,27]. CK17-expression was also increased in de-VIL and HSIL, compared to non-dysplastic tissue. However, similarly to Podoll et al. [27], we also observed that CK17-expression can be higher in dVIN than in HSIL. This could be due to the paradoxical maturation exhibited by the dysplastic cells of dVIN, as it has been reported that CK17-expression is most prominent in dysplastic cells with some degree of phenotypic maturation [53].

For SOX2, increased expression was observed in dVIN showing mutant patterns or wild-type p53-expression, compared to non-dysplastic vulvar tissue, in line with the previous report [29]. Increased SOX2-expression was also observed in de-VIL and HSIL, compared to non-dysplastic tissue. However, unlike CK17, SOX2-expression levels were fairly similar in dVIN and HSIL, as was also reported by Brustmann et al. [29].

For both CK17 and SOX2, we sought to identify lesion-specific expression patterns, as pattern-based IHC-interpretation can be more reproducible and easier to implement in research or practice, than manual counting of stained cells [30]. Diffuse, moderate-to-strong expression of CK17 or SOX2, across full epithelial thickness, or in the basal and suprabasal layers showed high sensitivity and specificity for the diagnosis of dVIN. Among p53, CK17, and SOX2, the highest sensitivity was obtained for SOX2 (86%), whereas, the highest specificity was obtained for p53 (83%) (Table S1). We therefore believe that CK17 and SOX2, when used in a panel along with p53, can complement the pathological assessment of dVIN. However, CK17 and SOX2, cannot be used to discriminate dVIN from HSIL. Moreover, for both CK17 and SOX2, increased expression was observed in a number of non-dysplastic lesions, whereas, a number of dVIN showed minimal expression. Therefore, for discriminating dVIN from non-dysplastic lesions, CK17 and SOX2 should be interpreted in the context of histology and clinical information, and should not be used as the sole diagnostic criterion. Further studies may help determine whether non-dysplastic lesions that show CK17 and SOX2 expression harbor molecular perturbations associated with early pre-neoplastic changes that precede histological manifestation.

In this study, we also investigated the molecular profile of a subset of dVIN and de-VIL, with a view to facilitate the identification of other potential diagnostic markers. In dVIN, *TP53* and *CDKN2A* mutations were detected most frequently, in line with previous reports [47,54,55,56,57,58,59]. Interestingly, *TP53* mutations were also detected in de-VIL, and dVIN that showed wild-type-expression on p53-IHC. Discordant results of *TP53* mutations and p53-IHC have been previously reported for dVIN [22]. Akbari et al. also detected *TP53* mutations of uncertain significance in some of the de-VIL in their cohort [24]. The higher frequency of *TP53* mutations in our cohort could be explained by the greater sequencing depth of our NGTS panel, which covers all exonic sites of *TP53*. In de-VILs, we could detect *PIK3CA* mutations, which have been reported to characterize these lesions [23,24]. However, these were not present in all de-VILs. Other interesting findings from the NGTS were: (i) frequent detection of *RNF43* mutations in both dVIN and de-VIL, which have been previously associated with endometrial carcinoma [60], and (ii) detection of mutations in *KEAP1*, *CDH1*, *PIK3R1,* and *POLE* exclusively in de-VIL. We also observed that a large number of the mutated genes in both dVIN and de-VIL could be mapped to the PI3K/AKT/mTOR pathway [61], indicating a potential involvement of this pathway in HPV-independent VSCC carcinogenesis. Nevertheless, to determine the clinical significance of these preliminary findings, further investigation in larger cohorts is necessary.

There are several limitations associated with the current study. As this is a retrospective, single-center study, a selection bias cannot be ruled out. Our findings need to be validated in prospective, multi-center cohorts. The IHC-markers were assessed by visual estimation, and manual assessment is always associated with a certain degree of subjectivity. Computerized image analyses may offer higher accuracy and help identify more reliable cut-offs to improve the interpretation of CK17 and SOX2 IHC. Furthermore, our NGTS panel comprised a limited number of amplicons, and the detections of mutations can be influenced by the platform used.

## 4. Materials and Methods

This retrospective, observational study follows the Standards for Reporting Diagnostic Accuracy (STARD) guidelines [62]; the checklist is provided in Table S3.

### 4.1. Histology Review

Vulvar lesions from 2014–2017 were identified from the electronic records of the Department of Pathology, Erasmus MC, and their hematoxylin-eosin (HE) stained glass slides were retrieved from the archives. These slides were reviewed by two pathologists (SDG and PCEG), and the lesions were classified as VSCC, dVIN, HSIL, de-VIL, or non-dysplastic vulvar tissue, using diagnostic criteria from literature [3,9,11,12,13,20,23,24,46,47,63]. Clinical data (age and treatment information) were gathered from the patient records. All patient data were anonymized and patient materials were handled following the guidelines of World Medical Association Declaration of Helsinki.

### 4.2. Immunohistochemistry (IHC)

For IHC, a set of dVIN, HSIL, de-VIL, and non-dysplastic vulvar cases having sufficient tissue to prepare serial sections was selected. To ensure a comprehensive study of the IHC-expression, the selection was prepared in a way to include—(i) lesions on vulvar skin with or without adnexal structures; (ii) standalone lesions, and lesions in tissue adjacent to VSCC; and (iii) lesions representing a range of histological appearances for each diagnosis. Cases with a history of chemo/radiotherapy were excluded.

Formalin-fixed paraffin embedded (FFPE)-tissues of the selected lesions were retrieved from the archives, and 4-µm-thick serial sections were prepared. For all lesions, IHC with p53 (clone: Bp53-11), p16 (CintecR^©^), dual stain CK17-MIB1 (Sp95-Ki67), and SOX2 (Sp67) was performed using an automated and validated staining system (Ventana Benchmark ULTRA, Ventana Medical Systems, Tucson, AZ, USA). The IHC protocol is detailed in Table S4.

For each lesion, the areas most representative of the diagnosis were marked on the HE-slides, so that identical areas could be studied on the IHC-slides. IHC was scored by SDG and PCEG by visual estimation, as described below.

p16: *block-type expression* = continuous, strong, nuclear and/or cytoplasmic expression involving ≥ 1/3rd of epithelial thickness [41]; *non-block type expression* = patchy expression in clusters of cells; *no expression* = complete lack of expression.

p53: *mutant patterns* = diffuse (basal to para-basal) overexpression/basal overexpression/null-pattern/cytoplasmic expression; *wild-type pattern (scattered)* = scattered, heterogeneous, basal or para-basal expression; *wild-type pattern (mid-epithelial)* = heterogeneous, mid-epithelial expression with sparing of basal cells and/or lower parabasal cells [16,17,18].

MIB1: *increased expression* = increased nuclear expression in the basal and/or parabasal layers; *not increased expression* = sporadic nuclear expression in basal (minor component) layers and/or parabasal layers (major component) [30,39].

CK17 and SOX2: The following parameters were recorded—(i) percentage of cells showing uniform cytoplasmic (CK17) or nuclear (SOX2) expression, irrespective of the intensity (ii) intensity of expression (weak/moderate/strong), and (iii) distribution of expression within the epithelium. In case of variation in the intensity and/or distribution, the predominant pattern was scored.

### 4.3. Next Generation Targeted Sequencing (NGTS)

NGTS was performed on a subset of dVIN and all de-VIL. The methodology is detailed in Table S5. In brief, areas having minimum 50% lesional cells were selected, and micro-dissected manually from hematoxylin-stained slides into 5% Chelex 100 resin (BioRad Laboratories, Hercules, CA, USA) cell lysis solution (Promega, Madison, WI, USA). Tissue fragments were subjected to proteinase K digestion for 16 h at 56 °C. After inactivating proteinase K, and removing cell debris and Chelex resin by centrifugation, the extracted DNA was used without further purification. DNA-concentration was measured with Qubit 2.0 fluorometer (Thermo Fisher Scientific, Waltham, MA, USA).

For NGTS, a custom-made panel, designed using the AmpliSeq designer (Thermo Fisher Scientific, Waltham, MA, USA) was used. This panel comprises 1042 amplicons covering hotspot regions in 65 cancer-related genes, and single nucleotide polymorphisms (listed in Table S5). NGTS was performed using the Ion Torrent platform (Thermo Fisher Scientific) following manufacturer’s protocols. Ion AmpliSeq Library Kitplus–384 LV was used for library preparation.

Sequence information was analyzed with Variant Caller v.5.10.0.18 (Thermo Fisher Scientifc), and variants were annotated in a local Galaxy pipeline using ANNOVAR (version 2014-11-12, Wang Genomics Lab, University of Southern California, Los Angeles, CA, USA). For reporting, variants existing in Exome Variant Server NHLBI GO Exome sequencing project (ESP), the 100,000 Genomes Project, Genome Aggregation Database (GnomAD), or Genome of the NL (GoNL) databases were filtered out, and those having a variant allele frequency > 10%, and located in the exons or splice sites were included (with the exception of *TERT*-promoter mutations). Pathogenicity of coding non-synonymous variants were predicted using Protein Variation Effect Analyzer (PROVEAN), Sorting Tolerant From Intolerant (SIFT), and UMD-Predictor algorithms, and the Catalogue of Somatic Mutations in Cancer (COSMIC).

### 4.4. Ethics Statement

This study follows the guidelines of the Dutch Federation of Biomedical Scientific Societies (www.federa.org/codes-conduct, accessed on 15 March 2021), which state that no separate ethical approval is required for the use of anonymized residual tissue procured during regular treatment.

### 4.5. Statistical Analyses

Data were analyzed using SPSS Statistics (Version 25.0: IBM Corp., Armonk, NY, USA). Independent sample’s *t*-test was used to compute two-sided *p*-values and identify statistically significant differences. *p*-value < 0.05 was considered statistically significant. Receiver operating characteristic (ROC) curves were plotted for p53, CK17, and SOX2, and areas under the curve (AUC) with 95% confidence intervals (CI) were measured, to assess their individual sensitivity and specificity for the diagnosis of dVIN. Sensitivity, specificity, positive predictive value (PPV), and negative predictive value (NPV) of p53, CK17, and SOX2 for the diagnosis of dVIN were computed, using the histological diagnoses as gold-standard. Expression levels of CK17 and SOX2 were visualized by constructing bar-charts and box-plots. NGTS data were analyzed descriptively.

## 5. Conclusions

CK17 and SOX2 show significantly higher expression in dVIN that show mutant pattern or wild-type p53-expression, compared to non-dysplastic vulvar tissues. Particularly for lesions where dVIN is suspected on histology, and p53-expression is wild-type, diffuse, strong expression of CK17 and SOX2 can help confirm the diagnosis of dVIN. Therefore, we infer that immunohistochemical markers CK17 and SOX2 can be useful adjuncts to p53 for the diagnosis of dVIN.

## Figures and Tables

**Table 1:** Clinico-pathological characteristics.

	Mean Age (95% CI)	Adjacent *	Standalone	Vulvar Skin with Adnexa	Vulvar Skin without Adnexa
		Number of Cases (Percentage)			
dVIN (*n* = 56)	69.3 (66.0–72.8)	35 (63)	21 (38)	37 (66)	19 (34)
de-VIL (*n* = 8)	68.3 (58.9–77.6)	4 (50)	4 (50)	6 (75)	2 (25)
HSIL (*n* = 27)	64.2 (57.8–70.5)	8 (30)	19 (70)	24 (89)	3 (11)
Non-dysplastic vulvar tissue (*n* = 46)	63.2 (58.2–68.2)	23 (50)	23 (50)	38 (83)	8 (17)

dVIN: differentiated vulvar intraepithelial neoplasia; deVIL: differentiated exophytic vulvar intraepithelial lesion; HSIL: high grade squamous intraepithelial lesion; * Adjacent to VSCC.

**Table 2:** Immunohistochemical expression of p53, p16, CK17, SOX2, and MIB1.

Immunohistochemical Marker		Expression Patterns	Diagnoses			
			dVIN (*n* = 56)	de-VIL (*n* = 8)	HSIL (*n* = 27)	Non-Dysplastic Vulvar Tissue (*n* = 46)
			Number of Cases (Percentage)			
p53	mutant patterns	Parabasal/diffuse overexpression	30 (54)	0 (0)	0 (0)	1 (2)
		Basal overexpression	1 (2)	0 (0)	0 (0)	7 (15)
		Null-pattern	10 (18)	0 (0)	0 (0)	0 (0)
	wild-type patterns	Wild-type (scattered)	15 (26)	8 (100)	1 (4)	38 (83)
		Wild-type (mid-epithelial)	0 (0)	0 (0)	26 (96)	0 (0)
p16		Block-type expression	0 (0)	0 (0)	26 (96)	0 (0)
		Non-block-type expression	2 (4)	0 (0)	1 (4)	7 (15)
		No expression	54 (96)	8 (100)	0 (0)	39 (85)
MIB1		Increased expression	29 (52)	7 (88)	27 (100)	18 (39)
		Not increased expression	27 (48)	1 (12)	0 (0)	28 (61)
CK17	Diffuse, moderate-strong, full epithelial expression		27 (48)	5 (63)	6 (22)	1 (2)
	Diffuse, moderate-strong, suprabasal expression		18 (32)	2 (25)	11 (41)	3 (7)
	Patchy, moderate-strong, suprabasal expression		8 (14)	1 (12)	10 (37)	5 (11)
	Patchy, weak, suprabasal expression		2 (4)	0 (0)	0 (0)	19 (41)
	No expression		1 (2)	0 (0)	0 (0)	18 (39)
SOX2	Diffuse, moderate-strong, full epithelial expression		37 (66)	5 (63)	12 (44)	2 (4)
	Diffuse, moderate-strong, basal and suprabasal expression		11 (20)	2 (25)	12 (44)	7 (15)
	Scattered, weak, basal (predominant) and suprabasal expression		7 (13)	1 (12)	2 (8)	17 (37)
	No expression		1 (1)	0 (0)	1 (4)	20 (44)
**Median percentage of cells showing expression (95% CI)**						
CK17			65 (55.3–69.4)	90 (53.7–95)	45 (27.6–50.2)	5 (14.4–32.1)
SOX2			75 (61.8–76.1)	78 (48–96.9)	80 (58.4–81.5)	5 (12.8–29.2)

dVIN: differentiated vulvar intraepithelial neoplasia; de-VIL: differentiated exophytic vulvar intraepithelial lesion; HSIL: high grade squamous intraepithelial lesion.

## Data Availability

All relevant data has been provided in the Supplementary Materials.

## Supplementary Materials

The following are available online at https://www.mdpi.com/article/10.3390/ph14040324/s1, Table S1: Test characteristics of p53, CK17, and SOX2 immunohistochemistry, Table S2: List of mutations detected with nest generation targeted sequencing, Table S3: STARD checklist, Table S4: Immunohistochemistry (IHC) protocol; Table S5: Tissue processing, DNA-isolation, and list of amplicons in the next generation targeted sequencing panel.
