# Supplementary material for: Evaluation of Immunohistochemical Markers, CK17 and SOX2, as Adjuncts to p53 for the Diagnosis of Differentiated Vulvar Intraepithelial Neoplasia (dVIN)
Source: Pharmaceuticals (Basel). 2021 Apr 2;14(4):324. doi: 10.3390/ph14040324 (PMC8066509; doi:10.3390/ph14040324)
Supplement: Supplementary file 1 [file pharmaceuticals-14-00324-s001.zip › Table S1_Test characteristics.docx]

# **Table S1**

**Test characteristics of p53, CK17, and SOX2 immunohistochemistry**

|  | **Sensitivity (95% CI)** | **Specificity (95% CI)** | **PPV (95% CI)** | **NPV (95% CI)** | **Accuracy (95% CI)** |
| --- | --- | --- | --- | --- | --- |
| **p53** | 74 (64.3 – 82.3) | 83 (73.4 – 89.5) | 81 (73.6 – 87.2) | 76 (68.6 – 81.3) | 78 (71.8 – 83.7) |
| **CK17** | 80 (70.8 – 87.3) | 80 (70.8 – 87.3) | 80 (72.8 – 85.7) | 80 (72.8 – 85.7) | 80 (73.8 – 85.3) |
| **SOX2** | 86 (77.6 – 92.1) | 81 (71.9 – 88.2) | 82 (74.9 – 87.2) | 85 (77.9 – 90.5) | 84 (77.6 – 88.4) |

*CI: confidence intervals
