# Supplementary material for: Evaluation of Immunohistochemical Markers, CK17 and SOX2, as Adjuncts to p53 for the Diagnosis of Differentiated Vulvar Intraepithelial Neoplasia (dVIN)
Source: Pharmaceuticals (Basel). 2021 Apr 2;14(4):324. doi: 10.3390/ph14040324 (PMC8066509; doi:10.3390/ph14040324)
Supplement: Supplementary file 1 [file pharmaceuticals-14-00324-s001.zip › Table S4_IHC protocol.docx]

# **Table S4**

**Immunohistochemistry (IHC) protocol**

For IHC, serial sections of 4 μm thickness were prepared from formalin fixed paraffin embedded (FFPE) tissues, and were mounted on adhesive glass slides. Immunohistochemistry was performed using an automated, validated and accredited staining system (Ventana Benchmark ULTRA, Ventana Medical Systems, Tucson, AZ, USA).

De-paraffinization was performed according to the BenchMark Ultra protocol. Antigen retrieval was performed using CC1 antigen retrieval solution (ref. 950-124, Ventana Medical Systems, Inc) for 64 minutes. For detection, UltraView universal DAB (ref. 760-500, Ventana Medical Systems, Inc.) or Ultraview Universal Alkaline Red detection kit (ref. 760-501) were used. Next, the specimens were incubated with the respective primary antibodies. Amplification was performed using Kit ref: 760-080. This was followed by counterstaining with haematoxylin II (ref: 790-2208, Ventana Medical Systems, Inc). Each slide contained an appropriate positive control. Details regarding the primary antibodies, detection, and amplification are tabulated below:

| **Antibodies** | **Clone** | **Supplier** | **Reference number** | **Detection** | **Incubation with primary antibody** | **Amplification** |
| --- | --- | --- | --- | --- | --- | --- |
| CintecR^©^ p16 | -- | Ventana | 805-2020 | UltraView DAB | 37°C for 12 min. | Yes |
| p53 | Bp53 -11 | Ventana | 760-2542 | UltraView DAB | 37°C for 4 min. | Yes |
| SOX2 | Sp67 | Cell Marque | 760-4621 | Ultraview DAB | 37°C for 60 min. | No |
| CK17 | Sp95 | Ventana | 790-4560 | Ultraview Red | 37°C for 20 min. | No |
| MIB1 | Ki67 | Ventana | 790-4286 | Ultraview DAB | 37°C for 28 min. | No |
