# Supplementary material for: Evaluation of Immunohistochemical Markers, CK17 and SOX2, as Adjuncts to p53 for the Diagnosis of Differentiated Vulvar Intraepithelial Neoplasia (dVIN)
Source: Pharmaceuticals (Basel). 2021 Apr 2;14(4):324. doi: 10.3390/ph14040324 (PMC8066509; doi:10.3390/ph14040324)
Supplement: Supplementary file 1 [file pharmaceuticals-14-00324-s001.zip › Table S5_Tissue processing, DNA-isolation, and list of amplicons.docx]

# **Table S5**

1. **Methodology of tissue processing and DNA-isolation for next generation targeted sequencing**

For DNA-isolation, eight consecutive 5µm-thick sections were prepared from formalin-fixed paraffin embedded tissues. These were next de-paraffinized and stained with hematoxylin, prior to microdissection. Lesional areas having minimum 50% tumor cells were selected, and micro-dissected manually from hematoxylin-stained slides using a sterile scalpel. These were added to 5% Chelex 100 resin (BioRad Laboratories, Hercules, CA) cell lysis solution (Promega, Madison, WI, USA). Tissue fragments were subjected to proteinase K digestion for 16 hours at 56 °C. Proteinase K was inactivated at 95 °C for 10 min. Next, the samples were centrifuged at 20,000 g for 5 minutes to remove the remaining cell debris and Chelex resins. The extracted DNA was collected in new tubes and stored at −80 °C until further use. DNA-concentration was measured with Qubit 2.0 fluorometer (Thermo FisherScientific, Waltham, MA, USA), and the extracted DNA was used without further purification.

1. **List of amplicons included in the next generation targeted sequencing panel**

| **Coding sequences – coverage** | |
| --- | --- |
| *ARID1A* | 100% |
| *BAP1* | 100% |
| *CDH1* | 100% |
| *CDKN2A* | 100% |
| *KEAP1* | 100% |
| *PIK3R1* | 100% |
| *PTEN* | 100% |
| *RB1* | 99% |
| *STK11* | 100% |
| *TP53* | 100% |
| *VHL* | 100% |
| **Mutation hotspots** | |
| *AKT1* | 3 |
| *AKT2* | 3 |
| *AKT3* | 2 |
| *ALK* | 20, 22-25 |
| *APC* | 16 |
| *ARAF* | 7 |
| *BRAF* | 11, 12, 14, 15 |
| *CDK4* | 2, 4, 7, 8 |
| *CTNNB1* | 3, 7, 8 |
| *DDR2* | 14-19 |
| *EGFR* | 12, 18-21 |
| *EIF1AX* | 1, 2 |
| *HER2* | 8, 17-21 |
| *ERBB3* | 3, 6-10, 21, 23 |
| *ESR1* | 4, 5, 7, 8 |
| *EZH2* | 16 |
| *FBWX7* | 9, 10 |
| *FGFR1* | 4, 7, 12-14 |
| *FGFR2* | 7, 9, 12 |
| *FGFR3* | 7, 9, 14, 15 |
| *FOXL2* | 1 |
| *GNA11* | 4, 5 |
| *GNAQ* | 4, 5 |
| *GNAS* | 8, 9 |
| *HRAS* | 2-4 |
| *IDH1* | 4 |
| *IDH2* | 4 |
| *JAK2* | 14 |
| *JAK3* | 4, 16 |
| *KIT* | 8, 9, 11, 13-18 |
| *KNSTRN* | 1 |
| *KRAS* | 2-4 |
| *MAP2K1* | 1-6 |
| *MET* | 2, 14, 19, 20 |
| *MTOR* | 30, 39, 40, 43, 47, 53, 56, 57 |
| *MYD88* | 5 |
| *NFE2L2* | 2 |
| *NOTCH1* | 26, 27 |
| *NRAS* | 2-4 |
| *OXA1L* | 1 |
| *PDGFRA* | 12, 14, 18 |
| *PIK3CA* | 2, 5, 8, 10, 14, 21 |
| *POLD1* | 6, 8, 12, 15-17, 24 |
| *POLE* | 9-14, 21, 25 |
| *RAC1* | 2 |
| *RAF1* | 7 |
| *RET* | 11, 16 |
| *RHOA* | 2 |
| *RIT1* | 4, 5 |
| *RNF43* | 2-10 |
| *ROS1* | 36-41 |
| *SF3B1* | 14, 15 |
| *SMAD4* | 3, 9, 12 |
| **Non-coding sequence** | |
| *TERT* (promoter) | |
| **Copy number variation on the following loci** | |
| 1p, 3p, 5q, 6p, 7, 8p, 9p, 10q, 11q, 13q, 15q, 16q, 17, 18q, 19 | |
